# A ‘wiring diagram’ for source strength traits impacting wheat yield potential

**DOI:** 10.1093/jxb/erac415

**Published:** 2022-11-05

**Authors:** Erik H Murchie, Matthew Reynolds, Gustavo A Slafer, M John Foulkes, Liana Acevedo-Siaca, Lorna McAusland, Robert Sharwood, Simon Griffiths, Richard B Flavell, Jeff Gwyn, Mark Sawkins, Elizabete Carmo-Silva

**Affiliations:** Division of Plant and Crop Science, School of Biosciences, University of Nottingham, Sutton Bonington LE12 5RD, UK; International Maize and Wheat Improvement Center (CIMMYT), Km. 45, Carretera Mexico-Veracruz, El Batan, Texcoco, Mexico; Department of Crop and Forest Sciences, University of Lleida–AGROTECNIO-CERCA Center, Av. R. Roure 191, 25198 Lleida, Spain; ICREA (Catalonian Institution for Research and Advanced Studies), Barcelona, Spain; Division of Plant and Crop Science, School of Biosciences, University of Nottingham, Sutton Bonington LE12 5RD, UK; International Maize and Wheat Improvement Center (CIMMYT), Km. 45, Carretera Mexico-Veracruz, El Batan, Texcoco, Mexico; Division of Plant and Crop Science, School of Biosciences, University of Nottingham, Sutton Bonington LE12 5RD, UK; Hawkesbury Institute for the Environment, Western Sydney University, Richmond NSW 2753, Australia; John Innes Centre, Norwich Research Park, Colney Ln, Norwich NR4 7UH, UK; International Wheat Yield Partnership, 1500 Research Parkway, College Station, TX 77843, USA; International Wheat Yield Partnership, 1500 Research Parkway, College Station, TX 77843, USA; International Wheat Yield Partnership, 1500 Research Parkway, College Station, TX 77843, USA; Lancaster Environment Centre, Lancaster University, Lancaster LA1 4YQ, UK; MPI of Molecular Plant Physiology, Germany

**Keywords:** Biomass, breeding, photosynthesis, source–sink, yield physiology

## Abstract

Source traits are currently of great interest for the enhancement of yield potential; for example, much effort is being expended to find ways of modifying photosynthesis. However, photosynthesis is but one component of crop regulation, so sink activities and the coordination of diverse processes throughout the crop must be considered in an integrated, systems approach. A set of ‘wiring diagrams’ has been devised as a visual tool to integrate the interactions of component processes at different stages of wheat development. They enable the roles of chloroplast, leaf, and whole-canopy processes to be seen in the context of sink development and crop growth as a whole. In this review, we dissect source traits both anatomically (foliar and non-foliar) and temporally (pre- and post-anthesis), and consider the evidence for their regulation at local and whole-plant/crop levels. We consider how the formation of a canopy creates challenges (self-occlusion) and opportunities (dynamic photosynthesis) for components of photosynthesis. Lastly, we discuss the regulation of source activity by feedback regulation. The review is written in the framework of the wiring diagrams which, as integrated descriptors of traits underpinning grain yield, are designed to provide a potential workspace for breeders and other crop scientists that, along with high-throughput and precision phenotyping data, genetics, and bioinformatics, will help build future dynamic models of trait and gene interactions to achieve yield gains in wheat and other field crops.

## Teaser

We summarize and update the origins of and limitations to sources of photosynthate in the context of a ‘wiring diagram’, depicting source and sink within wheat growth and development.

## Introduction: The complexities of wheat source and source–sink interactions and the need for a ‘wiring diagram’

A simplistic description of plant functions may be presented in terms of source and sink (Mason and Maskell, 1928; Martínez-Vilalta *et al.*, 2016; Chang *et al.*, 2017) in which a source organ is a net generator of a resource such as reduced carbon (e.g. sucrose) or reduced nitrogen (N; e.g. amino acids) and moves/exports this to a sink which is defined as a net consumer or storer of the material. Plant growth is then dependent on having both sufficient source and sink activities, which are interdependent. Most commonly, a photosynthetic leaf is viewed as a source, exporting sucrose to distant developing organs. However, any part of the plant that requires net import can act as a sink during development, such as grain, fruit, expanding leaves, and roots. It is also possible for organs to re-export resources that were previously received. An example is stem tissues in cereals, which act as temporary reserves of carbohydrates, or a senescing leaf exporting amino acids derived from chloroplasts.

Simplifying things, for wheat yield determination, the main source ‘players’ are the photosynthesizing organs (leaves and spikes), while the sinks are the developing florets/grains, and the stems play a dual role as major sinks before grain filling (during their own growth but also whilst storing carbohydrate) and change role to become a significant source afterwards when stored reserves are remobilized. Roots are also important players in source–sink interactions, behaving as sinks for carbon but may also be viewed as sources of other minerals taken up from the soil. In this review, we consider carbon as the ‘currency’: there are clear interactions with other resources such as N but these are beyond the scope of this review. Sources and sinks interact strongly in whole plants. First, source strength is needed to construct a sink with a large capacity to drive yield. On the other hand, inadequate sink size or activity can also limit source capacity via feedback mechanisms (for a review, see White *et al.*, 2016). If yields are to be increased, especially in species with a high harvest index, it is essential that the capacity of the source is optimized for the sink, and vice versa. However, interactions occur continuously between multiple sinks and sources. These, together with the influence of variable environmental conditions on metabolism, make the analysis and quantification of source–sink dynamics complex, for example when comparing variety differences. Whilst past gains in wheat have been achieved by increasing sink strength, source strength is the important limiting factor supporting grain set and essential for sink establishment (Reynolds *et al.*, 2022). The power of the source at any one moment is typically quantified via established methods of measurements of leaf area and photosynthesis assisted by modelling. There is no equivalent type of methodology for sink activity. Harvest index (the proportion of plant biomass formed by the harvested grain) or the number of grains per unit aboveground dry matter are probably our best proxies for sink strength (Chang *et al.*, 2017; Smith *et al.*, 2018).

When characterizing source activity, both light interception and the conversion of intercepted solar energy to dry matter (radiation use efficiency or RUE) are important. Maximum RUE provides the ceiling value to primary productivity in terms of dry matter production under any condition. The photosynthesis rate is strongly linked to RUE. This is shown by plant species which have evolved CO_2_-concentrating mechanisms, such as C_4_ photosynthetic metabolism in which primary CO_2_ fixation is spatially or physically separated from carbon assimilation in the Calvin–Benson–Bassham cycle (CBBC), and have typically higher RUE in warm environments. However, empirical and theoretical evidence suggests that RUE in C_3_ plants is substantially below optimum in the field (Sinclair and Muchow, 1999; Zhu *et al.*, 2008, 2010), which provides cause for optimism for improving primary productivity for C_3_ crops in particular. The reasons for the losses in radiation conversion have been extensively analysed in studies of photosynthesis, photorespiration, photoprotection, and respiration (Murchie *et al.*, 2009; Zhu *et al.*, 2010; Ort *et al.*, 2015). The inefficiencies of Rubisco have been highlighted as being of particular importance as they are central to the higher RUE of C_4_ compared with C_3_ species (Carmo-Silva *et al.*, 2015). Moreover, proof of concept experiments using crop and model species have shown that targeted intervention and manipulation of photosynthetic processes can enhance biomass and yield with a known basis, through improvements to RUE (Kromdijk *et al.*, 2016; Hubbart *et al.*, 2018). Through increasingly sophisticated modelling, it is now possible to predict the impact of photosynthetic interventions in a target field environment (Wu *et al.*, 2019).

A ‘top-scale’ indicator such as RUE is useful to consider in the context of this review and its companion paper on sinks (Slafer *et al.*, 2023) because it is dependent on diverse processes including carbon transport limitations (sink feedback), respiration, photoprotection/photoinhibition, and root mass accumulation. Evidence exists for genetic variation affecting RUE during pre- and post-anthesis phases in wheat (Calderini *et al.*, 1997; Acreche and Slafer, 2009; Molero *et al.*, 2019). Whilst photosynthesis is a primary driver of RUE, it is highly sensitive to external environmental conditions and internal regulation. As we highlight below, the photosynthesis cannot be represented by a single rate but rather as a series of efficiencies occurring in a dynamic environment.

The origins of photosynthate are also structurally diverse: in the wheat plant, chloroplasts are found not only in leaf blades but also in the spike and in the leaf sheaths which together make an important contribution to yield (Molero and Reynolds, 2020; Rivera-Amado *et al.*, 2020). Moreover, leaves in the lower canopy have distinctive photosynthetic and photoprotective characteristics compared with those in the upper canopy (Townsend *et al.*, 2018; Foo *et al.*, 2020). The collective arrangement of chloroplasts responsible for the source is therefore complex and diverse within the plant and, as discussed below, has diverse regulatory states depending on location.

Key regulated components of plant growth are the development and operation of sinks (Slafer *et al.*, 2023). In wheat, they include the developing reproductive parts; that is, the spike and grain and the transient storage in the stem, as well as meristems supporting new growth above- and belowground and stems receiving and storing carbohydrates during the vegetative stage. Interactions between the source (which is itself complex), the timing of reproductive development, and the changing size and activity of various sinks creates a network of interactions that is not yet fully understood. The interactions between source and sink ultimately determine primary crop productivity and remain important targets for scientific discovery.

**Fig. 1.**
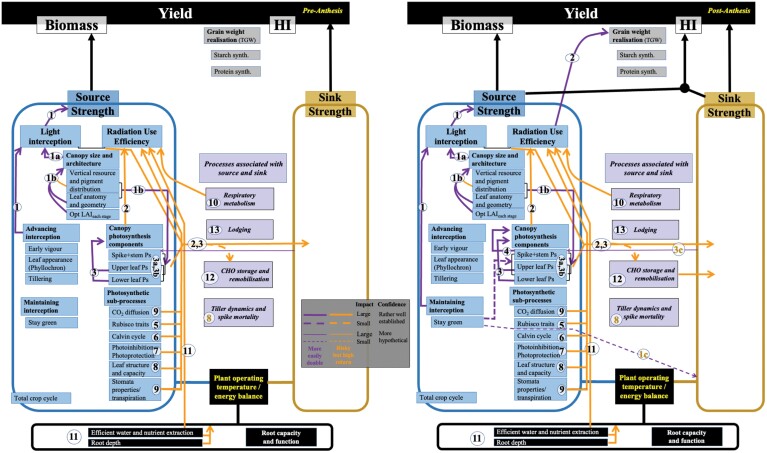
A wiring diagram for source generation in wheat at the pre-anthesis stage (left) and at the post-anthesis stage (right). The thickness of the wires reflects the extent of the evidence underpinning the link represented by the wire. The shape of the wire stands for the expected magnitude of impact on yield potential, and the colour of the wire reflects the ease/difficulty of managing the trait in breeding programmes (see inset). The number on each wire refers to the link which describes in the text the evidence behind each link. LAI, leaf area index; Ps, photosynthetic rate; Opt, optimal.

All of the above processes and interactions contribute dynamically to the amount of carbon that a crop stand accumulates in seeds. The efficiencies and interactions of the many processes influence both source and sink and the interactions between them, compounded by variable responses of components to the environment (Sanchez-Bragado *et al.*, 2020). The complexities and inter-relationships between source and sink processes and the need to optimize them in a whole-crop context have led to the concept of a ‘wiring diagram’ (WD) which links together all key processes underpinning yield potential according to developmental phase. This concept was broadly introduced in Reynolds *et al.* (2022) and is presented in greater detail therein and in the companion paper (Fig. 1; Slafer *et al.*, 2023). The series of WDs clarifies the key events responsible for yield potential as they occur during crop development, for example pre- and post-anthesis. This review analyses the diversity of individual source strength traits in wheat that underpin canopy photosynthesis. We present these traits within the WD of yield potential and then discuss the regulation of source activity by other yield potential-determining components integrated in the WDs. We consider carbon as the ‘currency’ in yield potential conditions: there are clear interactions with suboptimal conditions and other resources such as N, but these are beyond the scope of this review. Grain quality is an essential consideration, but it is also beyond our scope. In addition, genetic gains in CIMMYT spring wheat over 50 years of breeding appeared not to be at the expense of quality traits (Guzmán *et al.*, 2017).

## The relative importance of source strength within individual growth phases of wheat

A general expectation would be that improving leaf or chloroplast photosynthesis traits potentially enhances biomass production at all stages of development. As we discuss here, two major phases of functional significance can be identified, pre-anthesis (a yield construction phase when the crop source strength is used to build up structures determining the number and size potential of the grains, in turn responsible for yield potential) and post-anthesis (a yield realization phase when source strength is used to fill the grains, determining actual yield) (Sylvester-Bradley *et al.*, 2012). The leaf-level and chloroplast-level processes are relevant throughout, but may take on diverse roles according to their position in the canopy and canopy architecture which provide additional constraints such as self-shading and light fluctuations which become more relevant following the canopy closure phase.

The wiring diagram for the pre- and post-anthesis stages is shown in Fig. 1. The source strength components are shown in detail, in contrast to the sink traits which are described and defined in expanded detail in the companion paper (Slafer *et al.*, 2023). Several processes link source and sink biology and are important with regard to the regulation of both source and sink activities. These include respiration, stem storage of carbohydrates, tiller dynamics, signalling, and transfer of molecules between source and sink organs (Dong *et al.*, 2016; Posch *et al.*, 2019; Paul *et al.*, 2020). The links between components that are relevant to the improvement of yield are shown as wires with directional arrows in the WD and discussed in depth in the sections below. The wires within the WD are coded to impart more information with respect to the evidence defining their role and the ease with which the processes may be improved for wheat yield enhancement, as described in the legend to Fig. 1.

### Pre-anthesis (onset of stem extension to anthesis)

Photosynthesis drives crop growth up to anthesis, resulting in the construction of a canopy with an optimized leaf area index (LAI) for radiation capture. Early vigour, rapid tillering, and leaf appearance are critical for efficient canopy formation. Adequate photosynthate is necessary to advance light interception, promoting the development and rise in LAI to ensure construction of a canopy capable of delivering maximum light interception and photosynthesis during the critical stages for yield determination. A key growth stage at which maximum radiation interception and photosynthesis must commence is the onset of stem elongation. High canopy photosynthesis supports final grain number and grain weight potential, hence determining the final sink size (Slafer *et al.*, 2023). Consequently the timing of the source supply is important (Slafer *et al.*, 1994; Miralles *et al.*, 1998). Photosynthetic source supply is also necessary for the accumulation of stem storage carbohydrates, which are later remobilized to the grain according to the prevailing environmental conditions (Ruuska *et al.*, 2006). These stem water-soluble carbohydrates (WSCs) represent a strong and important sink for leaf photosynthate during the pre-anthesis phase. The requirement to supply stem storage while boosting the formation of structures determining sink strength during post-anthesis (i.e. grain number and potential grain size) highlights the importance of an adequate source supply during this phase (while highlighting a potential antagonism or trade-off between the two sink traits). Potential gene targets and single nucleotide polymorphisms (SNPs) associated with the size of the carbohydrate store have been described (Dong *et al.*, 2016).

### Post-anthesis (anthesis to maturity)

The emergence of the spike and anthesis mark a shift in source–sink dynamics in wheat. Canopy leaf senescence commences and WSC reserves may begin to be remobilized, the extent of which may depend on canopy photosynthesis. Therefore, grain filling is supported by photosynthesis in combination with the mobilization of the stem WSCs. If grain-filling conditions are not favourable for photosynthesis, the stem WSCs gain greater significance in terms of the final grain weight that is made up of pre-anthesis storage. Under high yield conditions, this can be minimal (Ruuska *et al.*, 2006). Additionally, it is increasingly recognized that spike and stem/sheath photosynthesis contribute significantly to grain weight during this phase (Molero and Reynolds, 2020). Maintenance of light interception through to the end of grain filling by optimized tiller dynamics and delayed senescence (stay-green trait) prolongs carbon assimilation, can potentially increase yield, and quantitative trait loci (QTLs) have been identified linked to such activities, but not in all cases (Spielmeyer *et al.*, 2007; Christopher *et al.*, 2018). However, the causal link between stay-green and yield is not clear since this is often considered a sink-limited phase (see Link 1c in Fig. 1). Under favourable conditions, when grain growth is co-limited during grain filling, this will be the situation because sink capacity may be limiting yield during early grain filling, and source capacity may limit it at later stages of grain filling (e.g. Acreche and Slafer, 2009).

## Individual source strength components

**Fig. 2.**
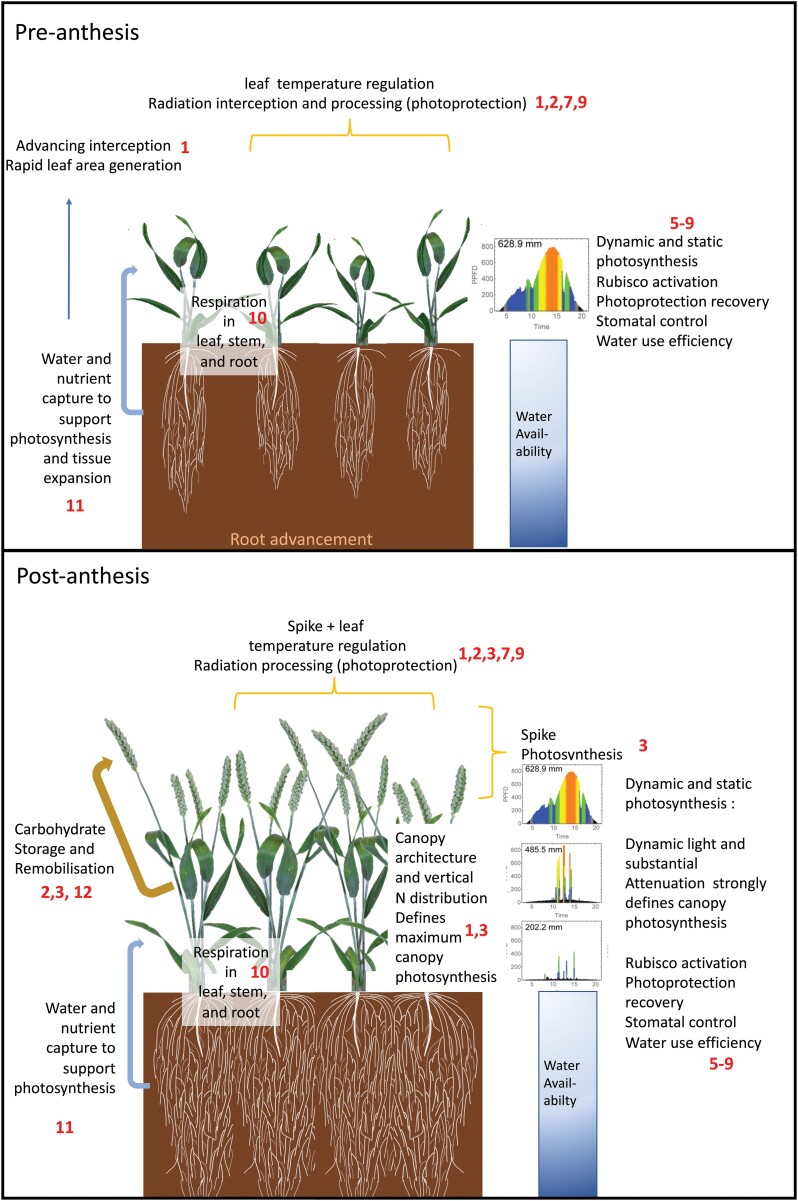
Whole-plant source characteristics and yield underpinning processes at two growth stages in wheat: pre-anthesis (top) and post-anthesis (bottom). Numbers in red refer to the links in Fig. 1.

In the WD (Fig. 1), the contributions of processes to higher order traits are numbered 1, 2, etc. Figure 2 illustrates the location and action of the different source components and processes within the canopy at two wheat growth stages.

### Canopy size and architecture, linked to light interception and radiation use efficiency (Links 1, 2, and 3)

The production of carbohydrate begins with the formation of a canopy to present a leaf surface area that captures solar energy for conversion. At early growth stages, rapid establishment and leaf expansion accelerate biomass production, and here light interception is considered to be of critical importance (Link 1). At such low leaf areas, less vertical orientation of foliage may provide an interception advantage and faster ground cover, and, as a result, full light interception is achieved relatively quickly. Crops are often sown at a density to maximize radiation interception during stem elongation, and growth during this phase is more relevant for yield determination than in earlier phases. It follows that much of the research on source productivity is focused on the efficiency of conversion rather than interception of absorbed radiation in high-yielding conditions. The origins of whole-canopy photosynthesis (considering here both radiation interception and conversion) are multiple. They include (i) canopy structure (given by the size and distribution of photosynthetically active organs, mainly leaf blades that make up the LAI, leaf sheaths covering the internodes, the last internode, the peduncle, and spikes); (ii) distribution of photosynthetic properties (within the plant and within the leaf); (iii) efficiency of individual components of photosynthesis within chloroplasts including light and dark reactions; and (iv) the functioning of associated processes such as respiration, stomatal behaviour, and transpiration capacity.

It has been established for many decades that light interception directly drives source strength, and hence biomass production, in a quantitative manner. The rapid generation and advancement of a large LAI (early vigour), via leaf appearance, and tillering can be important in some environments where the season is limited, and these properties may be associated with final yield. Reports of QTLs for early vigour exist (Botwright *et al.*, 2002). However early vigour may not be critical in yield potential systems where interception is not a limitation for much of the growing period, depending on correct planting density and agronomic practices.

Moving beyond interception, a photosynthetic canopy consists of the combined layers of vegetation within a stand of plants and has a three-dimensional structure which also changes over time, especially during the tillering and stem elongation phases. It is commonly assumed that in conditions where other resources are not limiting, an increase in photosynthesis can potentially drive a higher overall rate of plant growth, and this has in fact been demonstrated using diverse lines of evidence including free air CO_2_ enrichment (Cai *et al.*, 2016; Ainsworth and Long, 2021) and manipulation of the specific biochemical properties of leaves, for example by improving carboxylation efficiency and dynamic photoprotection (Kromdijk *et al.*, 2016; Głowacka *et al.*, 2018; Hubbart *et al.*, 2018; South *et al.*, 2018). However, a measurement of a single leaf at a single position (such as the light-saturated rate at ambient CO_2_ level), even at key growth stages, may not accurately predict whole-canopy carbon gain and yield. This is because this measure does not take into account diverse environmental conditions and also leaf positoning at different depths within the plant canopy where they are exposed to different microenvironments of temperature, light, CO_2_, and humidity (Link 3). This influences not only photosynthesis but also respiration. Leaf properties will also differ in terms of total N, Chl *a*:*b* ratio, and anatomy depending on position, age, and light acclimation status.

Canopy architecture influences productivity: a more upright canopy is thought to be more productive owing to additional opportunities for photon penetration and therefore a higher proportion of the canopy existing in a state closer to but not exceeding the light saturation point; that is, lower leaves are more productive and upper leaves avoid light saturation and photoinhibition (Long *et al.*, 2006; Song *et al.*, 2013; Burgess *et al.*, 2015; Richards *et al.*, 2019). Moreover, the environmental conditions within the canopy are frequently dynamic rather than static, especially in response to light intensity, sun angle, and temperature. In the pre-heading stage, tillering and stem extension create a highly dynamic leaf canopy architecture. Such variability can be accounted for within canopy photosynthesis models (of varying complexity) combined with empirical validation (Baldocchi and Amthor, 2001; Hirose, 2005; Zhu *et al.*, 2012; Burgess *et al.*, 2019; Chang *et al.*, 2022). In modelling and empirical architecture, studies of the contribution of lower leaves indicate that it is probably below potential. This is compounded by the knowledge that such leaves emerge into high light but become progressively shaded, limiting opportunities for low light acclimation (Murchie *et al.*, 2005; Robles-Zazueta *et al.*, 2021). Optimization of lower leaf biology, either by limiting their cost or by increasing their photosynthetic efficiency, would improve ‘return on investment’ of construction. Progress in understanding genetic variation in ‘below-canopy’ traits is dependent on high-throughput analysis (phenotyping). A current obstacle to such measurements is the large leaf area in an occluded location below the canopy surface, which is beyond the reach of most automated sensors and so requires manual analysis. Whilst still problematic for phenotyping, instrumentation and modelling to address lower canopy function is advancing (e.g. Burgess *et al.*, 2015; Taylor and Long, 2017; Wu *et al.*, 2019) (Link 3).

Wheat canopies are often densely packed, with light attenuated in the vertical direction according to the zenith and with varying proportions of scattered and direct radiation. The vertical distribution of irradiance leads to substantial acclimation effects. Since Rubisco and leaf N are closely related, this in turn leads to a common assumption that light, leaf N, and photosynthetic capacity should be correlated, which has been confirmed for many canopy types (Oguchi *et al.*, 2008). This has been extended to account for other functions of canopies such as N stores (e.g. for grain protein synthesis) and the interaction with fluctuating light (Hikosaka *et al.*, 2016; Townsend *et al.*, 2018). More recently, the physical properties of canopies that provide fluctuating and dynamic light to the leaves has generated the most interest (Kaiser *et al.*, 2018; Murchie *et al.*, 2018; Gibbs *et al.*, 2019). Solar positioning and wind-induced movement combined with complex 3D arrangements and multiple occlusions leads to a ‘4D’ pattern of light. This results in a constantly changing light intensity requiring a rapid photosynthetic and photoprotective response. These impact productivity and suggest that the way in which photosynthesis is regulated in response to fluctuations in the environment is a highly important determinant of plant productivity as well as its performance under steady-state or temporarily steady-state conditions. Light modelling such as ray tracing generates algorithms that are able to describe light dynamics in canopies (Song *et al.*, 2013; Wang *et al.*, 2017). These methodologies are useful but require refinement to account for canopy properties such as movement. Canopy models are able to utilize simple canopy representations either by making the assumption of a single or two-layer ‘leaf’ analogy or by utilizing more complex 3D representations that can handle the dynamics of photosynthesis using a ray tracing algorithm. Either way, the ability to model dynamic photosynthesis in a complex canopy with increasing realism is improving.

What are the possibilities for improving source generation? Both the size and architecture of the plant canopy (green area) determine the amount of radiation intercepted for ‘conversion’ into biomass. The critical maximum LAI or green area index (GAI; to include spikes and stems) enables the highest productivity and for a cultivar depends on leaf orientation, arrangement, and planting density, and typically can vary between 3 and 5 (Link 1b) (with 3 commonly considered as a minimum for a fully expanded canopy) (Foulkes and Murchie, 2011). Canopy size has been optimized for, and supports, interception during stem elongation, and the importance of a rapid establishment of critical LAI in early stages of growth is relevant to various extents depending on the growing conditions, chiefly the length of the growing season.

Architecture, as well as influencing optimal LAI, affects canopy conversion coefficients (i.e. RUE) by determining the penetration of light from upper leaves to the lower leaves and distribution of photosynthetic rates and efficiencies at various canopy positions (Link 1b). Modelling light transition and photosynthesis has shown that canopies with upright leaves have higher photosynthetic rates per unit absorbed radiation (Song *et al*., 2013, 2022) and reduced photoinhibition (Burgess *et al.*, 2015). Photosynthesis can be maintained close to the point of light saturation whilst reducing the proportion of light-supersaturated leaves. Recently, a study of two multiparent wheat populations showed that erectophile wheat canopies yielded 24% more grain than planophile canopies due to increased grain number and overall biomass production. Moreover, the same QTLs identified in this study were relevant in both dryland and irrigated environments (Richards *et al.*, 2019). Liu *et al.* (2018) also revealed strong reproducible QTLs within a different recombinant wheat inbred line population for flag leaf angle, length, area, and width, identifying potential targets for fine-mapping and marker-assisted selection.

The vertical distribution of pigments in a canopy is also of importance. Modelling and empirical data have shown that by reducing pigment concentration, especially in the upper leaves of a canopy, light can penetrate more efficiently to lower leaves which results in a distribution of photosynthetic activity provoking a greater canopy carbon gain (Walker *et al.*, 2018) (Link1b). Additionally, while the distribution of N through the canopy more or less mimics that of radiation (with more N allocated to upper layers and less to lower layers; Hirose and Werger, 1987; Drouet and Bonhomme, 1999), this distribution is considered suboptimal: the potential photosynthetic capacity of lower (shaded) leaves is in excess considering the low light they receive, even when high-intensity sunflecks are taken into consideration (and therefore an even lower N allocation to these leaves would in theory not reduce their actual photosynthesis), whilst upper leaves could increase their photosynthetic capacity if more N were allocated to them and this was invested in Rubisco (Townsend *et al.*, 2018). Therefore, there is likely to be room for improvement in relocating N (as photosynthetic components) to the upper parts of the canopy. Genetic variation in wheat for N distribution has been observed, but the underying basis of this has not been elucidated (Salter *et al.*, 2020).

Improvement of RUE itself is deemed possible due to the dominance of leaf and canopy photosynthesis in determining RUE and the recognition that photosynthesis operates below maximum efficiency (Zhu *et al.*, 2010). Since RUE is a culmination of all components of growth, improvement of RUE as a single trait is not often considered and QTLs are normally attributed to component processes. Field-level selection for RUE as a single trait will be an important target in future work (Furbank *et al.*, 2019) and RUE is clearly growth stage specific (Molero and Reynolds, 2020) with extant genetic variation and prevalence of source–sink interactions including the dynamics of temporary stem storage sinks. Root biomass formation will also co-determine RUE values and yet this is rarely taken into account. RUE is notoriously cumbersome to measure and is not a high-throughput trait. Despite its importance, the complex nature of RUE has meant that it has not been introduced as a routine trait for screening or breeding, although efforts are being made to develop remote and high-throughput measurement of RUE (Robles Zazueta *et al*., 2021).

### Foliar and non-foliar contributions to canopy photosynthesis (Links 3 and 4)

Leaf (and to a small extent stem) photosynthesis provides all of the photosynthate for a wheat plant prior to the formation of the reproductive spike (see below). The main features of canopy photosynthesis have been covered above. Measurements of the rate of leaf photosynthesis should ideally take into account context: the position in the canopy, the condition of the leaf under measurement, its environmental history, and age. Without these, any correlations between momentary steady-state measurements at light and CO_2_ saturation (*A*_max_) and biomass and yield are not necessarily expected. However, they are commonly found. There is ample evidence for variation in *A*_max_ among elite wheat lines (Driever *et al*., 2014, 2017), and photosynthesis measured at saturating light (ambient CO_2_) in flag leaves of field-grown winter and spring wheat before and after anthesis has been shown to be correlated positively with aboveground biomass and grain yield (Fischer *et al.*, 1998; Reynolds *et al*., 2000a, b; Gaju *et al.*, 2016). In some environments, and down through the canopy, plants might not experience a constant supply of saturating light conditions, thus the operating rate of photosynthesis at non-saturating light will contribute a large proportion of the photosynthate. Photosynthesis measured in flag leaves at ambient CO_2_ and a range of light intensities (especially non-saturating) before and after anthesis are positively correlated to grain yield, harvest index, and other photosynthetic traits such as the rates of electron transport (*J*_max_) and Rubisco activity (*V*_cmax_) (Carmo-Silva *et al.*, 2017; Lopez-Calcagno *et al*., 2020). The coordinated regulation of *J*_max_ and *V*_cmax_ during these phases is likely to be important to maximize operational photosynthesis. Flag leaf photosynthesis at booting contributes to define grain number, while post-anthesis it contributes to grain weight (as proposed by Faralli and Lawson, 2020). Therefore, static photosynthesis is an important trait to improve if it contributes to yield potential.

While the majority of photosynthetic research focuses on the leaves, the contribution of non-foliar photosynthesis has received much less attention. From cotton to cucumber, structures such as the stem, ripening fruiting bodies, bracts, and seeds have all demonstrated carbon uptake (Ishihara *et al.*, 1991; King *et al.*, 1998; Kong *et al.*, 2010; Hu *et al.*, 2012; Sui *et al.*, 2017; Furbank *et al.*, 2020; Henry *et al.*, 2020; Simkin *et al.*, 2020; Martinez-Pena *et al*., 2022). Limiting photosynthesis in these structures has a significant impact on yield. For example, Sanchez-Bragado *et al.* (2020) found that shading a wheat spike reduced spike grain weight and thousand kernel weight by ~40% and 27%, respectively. The potential for genetic variation in stem (peduncle) and sheath photosynthesis in contributing to grain yield has been shown (Rivero-Amado *et al*., 2020).

Located in a prominent position, and by definition present throughout grain filling, the wheat spike intercepts a high level of solar radiation (Sanchez-Bragado *et al.*, 2014a, b), experiencing little or no shading compared with the crowded canopy below. The spike under favourable conditions supports 20 or so spikelets, consisting of glumes, lemma, palea, and, sometimes, awns—a filament extension of the lemma. All these structures contain chlorophyll and stomata (Li *et al.*, 2017; Ding *et al.*, 2018; Simkin *et al.*, 2020), and therefore have the potential for gas exchange and photosynthetic carbon fixation (Tambussi *et al.*, 2007; Maydup *et al.*, 2012; Simkin *et al.*, 2020) in close proximity to the grain—the final sinks. Not only does this close proximity between source and sink allow for rapid translocation of carbohydrates but it also allows for the efficient re-fixation of respired CO_2_ from the developing kernel during grain filling (Bort *et al.*, 1996; Tambussi *et al.*, 2007). In addition, spike photosynthetic components—such as chlorophyll, Rubisco, and light-harvesting complex II (LHCII)—are retained in the spike relatively longer in comparison with the flag leaf, thereby sustaining higher photosynthetic efficiencies during grain filling under well-watered (Li *et al.*, 2006; Martinez-Pena *et al*., 2022) and drought-stressed conditions (Martinez *et al.*, 2003). Maintaining spike photosynthesis delays senescence, a target trait for improving yield, resulting in increased grain weight (Chapman *et al.*, 2021) and enhanced abiotic stress tolerance (Jagadish *et al.*, 2015).

The contribution of spike photosynthesis to grain filling has increased in line with the presence of *Rht* alleles (dwarfing alleles) over the years. This response is thought to be compensatory, with the spike contribution increasing with kernel number as crop height shortened and contributions of the stems declined (Maydup *et al.*, 2012; Wang *et al.*, 2016).

On an area basis and under well-watered conditions, wheat spike photosynthetic rates are lower than those observed in the leaf, although the area of the spike may be greater than that of the flag leaf (Tambussi *et al*., 2005, 2007; Zhou *et al.*, 2016) and the 3D structure of both make an area comparison difficult. However, the spike is estimated to supply 10–80% of photoassimilates to the grain and a 30–40% contribution to grain weight per spike (Molero and Reynolds, 2020), making this non-foliar organ a major source of photoassimilates for grain filling (Sanchez-Bragado *et al.*, 2020; Tambussi *et al.*, 2021) and a potential trait for selection. In addition, the spike demonstrates positive correlations between the rate of CO_2_ uptake and yield under contrasting environmental conditions (Inoue *et al.*, 2004; Molero and Reynolds, 2020), with the percentage contribution of the spike increasing under leaf source-limiting conditions (Maydup *et al*., 2010, 2014; Wang *et al.*, 2016) or when sink limitations are reduced (Sanchez-Bragado *et al.*, 2014b; Link 3c). The location of the spike means that it is exposed to high radiation—although their generally vertical angle reduces the photosynthetic photon flux density (PPFD)—and operates at a slightly warmer temperature than leaves (Ayenah *et al*., 2002) presumably because of limited cooling capacity. The stress biology of spikes and the role of awns have not been fully determined.

Direct measurement of net photosynthetic CO_2_ uptake of the spike should be interpreted cautiously, because changes in the rate of spike photosynthesis can be influenced by dark respiration (Sanchez-Bragado *et al.*, 2014a, b). Due to the high (and changing) rate of respiration which is associated with the high growth rate and a lack of knowledge of whether spike respiration rates vary between day and night, some researchers have chosen to calculate ‘gross photosynthesis’, the sum of net photosynthetic and dark respiration rates.

In summary, the spike is not simply a structure to support the development of the sink; growing research into spike photosynthesis highlights this complex inflorescence as a vital and complex source of photoassimilates for grain filling. Substantial genetic variation in spike photosynthesis has been reported across 196 lines and QTLs identified (Molero and Reynolds, 2020), and genetic variation has also been reported for leaf sheath photosynthesis (Rivera-Amado *et al.*, 2020). While among the lines studied, spike photosynthesis was not correlated with leaf photosynthesis—indicating independent genetic variation (Molero and Reynolds, 2020)—further work is needed to understand how photosynthesis in the spike differs from that in the leaves in response to changing environmental conditions, under abiotic stress, and as the plant ages. As pointed out by Martinez-Pena *et al*. (2022), non-foliar sources of photosynthate may have yield-forming roles at stages of growth or during environmental conditions where leaves are less able to contribute. Identifying spike photosynthetic traits, which maintain or improve source quantity or quality for grain formation and filling, will therefore be important for improving yields.

### Dynamic properties of photosynthesis: induction and relaxation (Links 5–9)

The photosynthetic rate is frequently in a state of change due to natural fluctuations in light, temperature, humidity, and other environmental factors (Kaiser *et al.*, 2018). Consequently, it cannot be assumed that photosynthesis is at steady state while in an agricultural or natural environment; this may be the exception rather than the rule. However, most research on photosynthesis in crop plants has been conducted within the context of momentary steady-state measurements where the number of fluxes entering the leaf are roughly equal to those exiting because they are the easiest to measure and interpret. The processes regulating the kinetics and coordination of photosynthesis in response to changes in light or other environmental factors are crucial in understanding how leaf photosynthesis can be scaled to the canopy level. These dynamic photosynthesis traits are an interplay between the slow induction and relaxation of key processes such as enzyme activation, photoprotection, and stomatal opening and closing (Kromdijk *et al.*, 2016; McAusland *et al.*, 2019; Acevedo-Siaca *et al*., 2020, 2021; Da Souza *et al*., 2022).

**Fig. 3.**
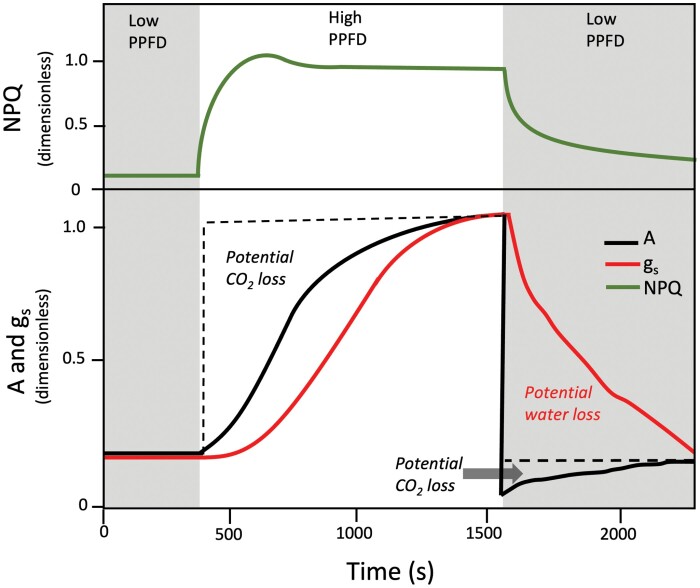
Schematic figure showing normalized temporal response of CO_2_ assimilation (A, black lines), stomatal conductance (g_s_, red lines), and non-photochemical quenching (NPQ, green lines) in wheat to an increase in photosynthetic photon flux density (PPFD) from ~120 (shaded area) to ~1000 (non-shaded area) and back to 120 µmol m^–2^ s^–1^ from a low-light-adapted state. When a leaf transitions from low PPFD to high PPFD, the rate of CO_2_ assimilation increases until potentially reaching a steady state, a process known as photosynthetic induction. The increase in stomatal conductance is much slower than the increase in CO_2_ assimilation, but even the latter is not immediate. Photosynthetic induction is characterized by a lag in photosynthetic efficiency relative to steady state, which can result in potential CO_2_ loss or forgone assimilation (area delineated by the dashed line) (see text for mechanisms of induction limitation). When a leaf moves from high PPFD to low PPFD, CO_2_ assimilation responds immediately but is accompanied by a slower stomatal response. This slower stomatal closure can result in potential water loss and decreased intrinsic water use efficiency. The photoprotective process NPQ is induced rapidly in high PPFD due to the action of PsbS and the synthesis of zeaxanthin but more slowly to relax back to its original value in part due to the slow conversion of zeaxanthin back to violaxanthin. In low light, the sustained presence of NPQ reduces the quantum yield of CO_2_ assimilation resulting in the loss shown (area delineated by the dashed line). This generalized schematic is based on known responses of C_3_ species.

The slow response of photosynthetic traits to changes in irradiance has been identified as a significant limitation to crop growth in a field setting (Carmo-Silva *et al.*, 2015; Kromdijk *et al.*, 2016; Taylor and Long, 2017; Kaiser *et al.*, 2018; Slattery and Ort, 2021). For example, photosynthetic induction—the increase in CO_2_ assimilation when a leaf is exposed to high light after a period of shade—is characterized by a lag in photosynthetic efficiency relative to steady-state photosynthesis (Fig. 3). A faster photosynthetic induction response, where leaves react more quickly to an increase in light, could result in plants with greater carbon assimilation and increased productivity (Taylor and Long, 2017; Acevedo-Siaca *et al.*, 2020). Meanwhile, during changes from high light to low light, slow stomatal kinetics and slow relaxation of non-photochemical quenching (NPQ) result in decreased water use efficiency and inefficient use of light at low light intensities, respectively (Kromdijk *et al.*, 2016; McAusland *et al*., 2016, 2020; Acevedo-Siaca *et al.*, 2021). Optimizing leaf responses to changes in light could lead to plants that also conserve more water, and with substantial within-species variation there is scope for improvement (McAusland *et al.*, 2016).

Past research has shown that an inefficient photosynthetic induction response in wheat may result in a biomass penalty of up to 21% (Taylor and Long, 2017). Additionally, significant variation has been found between wheat cultivars and its wild relatives during both photosynthetic induction and NPQ relaxation, with some landrace or wild germplasm outperforming elite varieties (McAusland *et al.*, 2020). These studies suggest that not only can these processes be improved in wheat, but that also there is significant natural variation that could be exploited. Additionally, key genes such as those encoding PsbS, zeaxanthin epoxidase, and violaxanthin epoxidase have been identified as possible routes to optimize response to change in light (Kromdijk *et al.*, 2016; Glowacka *et al.*, 2018; Kaiser *et al.*, 2018). It is expected that genes and outcomes such as these are likely to be conserved across species, and so knowledge from model plants and other crops could be directly transferable to wheat, with the caveat that limitations to non-steady-state photosynthesis can be species or even genotype dependent (Soleh *et al.*, 2016; De Souza *et al.*, 2020; Acevedo-Siaca *et al.*, 2020; Yamori *et al.*, 2020; Acevedo-Siaca *et al.*, 2021).

Furthermore, recent studies focusing on characterizing the dynamic properties of photosynthesis (largely in controlled conditions) suggest that we may need to reconsider the way in which photosynthesis is measured to more accurately reflect the field conditions in which crops are grown. It has been previously shown that more natural variation is seen between genotypes during non-steady-state conditions than steady-state conditions, suggesting that our previous understanding of natural variation for photosynthetic traits may be underestimated (Acevedo-Siaca *et al.*, 2020; McAusland *et al.*, 2020; Cowling *et al*., 2022). It remains difficult to measure dynamic properties at a high throughput required for field screening, for example by using gas exchange, solar-induced fluorescence, or spectral reflectance; however, this is an active research area (reviewed in Murchie *et al.*, 2018; Fu *et al.*, 2022). Recent advances in very high-throughput laboratory-based methodologies, for example using chlorophyll fluorescence (Ferguson *et al.*, 2020; McAusland *et al.*, 2020), have shown promise if these can be scaled to the field.

### Rubisco-linked traits (Link 5)

Rubisco plays a central role in carbon assimilation in all tissues, leaf and non-leaf, so it is a fundamental issue for crop improvement, and Rubisco is not a very efficient carboxylase enzyme. Here we summarize the key points for improvement of Rubisco activity in wheat which are likely to result not just in improved photosynthetic efficiency, and thereby productivity, but also in resource use efficiency, and thereby sustainability. Given the complexities of Rubisco function, progress has been relatively slow, but findings in the past 5–10 years suggest that the field is ripe to enhance measurable outputs in the near future.

One of the key limitations is that CO_2_ and O_2_ can both be used as gaseous substrates by the enzyme. Rubisco oxygenation leads to loss of previously fixed CO_2_ and NH_4_^+^ with energy expense during photorespiration. Substantial natural diversity exists in the CO_2_ fixation properties of higher plant Rubisco (Orr *et al.*, 2016; Sharwood, 2017; Sharwood *et al.*, 2022), including amongst wheat wild relatives (Prins *et al.*, 2016). This suggests that the catalytic diversity of plant Rubisco can be exploited in efforts to breed more productive wheat. The activity of Rubisco in response to environmental cues involves interaction with many cellular components and this regulation is not optimized for agricultural productivity (Carmo-Silva *et al.*, 2015). In addition, Rubisco could be made more responsive to natural fluctuations in environmental conditions: scope for ‘speeding’ up the rate of Rubisco induction in response to light exists and would lead to significant improvements in daily carbon assimilation (Taylor *et al.*, 2017).

The function of Rubisco can be optimized by tailoring its catalytic properties to the light and CO_2_ microenvironment at different positions in the canopy (Zhu *et al.*, 2004; Long *et al.*, 2006). While it would be advantageous to have high maximum carboxylation activity (*V*_cmax_) in illuminated leaves and spikes at the top of the canopy, in shaded leaves at the bottom of the canopy it would be best to have high Rubisco specificity towards the gaseous substrate CO_2_ relative to O_2_ (*S*_c/o_). These properties are likely to be determined by the chloroplast-encoded large and nuclear-encoded multigene small subunits of Rubisco (Martin-Avila *et al.*, 2020). Other proteins including specific sugar phosphate phosphatases are known to interact with Rubisco, and post-translational modifications may also play a role (Carmo-Silva *et al.*, 2015; Lobo *et al.*, 2019; Hayer-Hartl *et al.*, 2020). To enable engineering of improved Rubisco function in wheat canopies, identification of specific promoters and development of bioengineering tools (Alotaibi *et al.*, 2018; Belcher *et al.*, 2020; Cai *et al.*, 2020) are necessary to enable expression of different isoforms and proteins in leaves at the top and bottom of the canopy, as well as at different crop growth stages.

The assembly and abundance of Rubisco protein is determined by several protein chaperones and auxiliary factors involved in Rubisco biogenesis (Hayer-Hartl *et al.*, 2020). In wheat, Rubisco can represent >50% of the total soluble protein in the leaves (Carmo-Silva *et al.*, 2015). Decreasing the allocation of resources such as N to Rubisco (e.g. by making Rubisco more efficient and less abundant) could enable allocation of such resources to other limiting enzymes and result in increased yields (Reynolds *et al.*, 2012; Carmo-Silva *et al.*, 2015; Faralli and Lawson, 2020). The activity of Rubisco per N content in the leaf would be maintained as the overall activity of Rubisco is maintained, having less but more active enzyme. Variation in Rubisco activity per N (*V*_cmax25_/N) has been observed in the flag leaves of spring wheat grown under field and controlled conditions (Silva-Pérez *et al*., 2018, 2020), suggesting that natural diversity exists which could be exploited for improvement. Potential for using natural variation in Rubisco catalytic properties has been shown by modelling the replacement of Rubisco of *T. aestivum* with Rubisco from *Hordeum vulgare*, the wild *Aegilops cylindrica*, and maize in terms of achieving higher assimilation rates (Prins *et al.*, 2016; Sharwood *et al.*, 2016).

The speed of Rubisco activation in response to a shift from shade to fully illuminated conditions is regulated by Rubisco activase (*TaRca1* and *TaRca2*; Carmo-Silva *et al.*, 2015). Measurements of light induction of photosynthesis in flag leaves of glasshouse-grown wheat and subsequent modelling of the impact on diurnal carbon assimilation in light fluctuating environments showed scope for up to 21% assimilation gains associated with faster activation of Rubisco (Taylor and Long, 2017). Variation in Rubisco activase properties suggests scope for a bioengineering approach to speed up Rubisco activation (Perdomo *et al.*, 2019; Scafaro *et al.*, 2019). A breeding approach might also be possible since significant genetic variation in induction speed has been found amongst glasshouse-grown wheat (Salter *et al.*, 2019).

### Calvin–Benson–Bassham cycle (Link 6)

The rate of RuBP regeneration in the CBBC limits photosynthesis at high light and high CO_2_. Sedoheptulose-1,7-biphosphatase (SBPase) was identified as a limiting enzyme in this process (Poolman *et al.*, 2000; Lefebvre *et al.*, 2005; X.G. Zhu *et al.*, 2007). Genetically engineered wheat plants with increased expression and activity of SBPase in the vegetative stage showed higher photosynthesis at high light and high CO_2_, and increased biomass and grain yield under controlled conditions (Driever *et al.*, 2017). Variation in nature is insufficient to produce the levels of SBPase increase required (X.G. Zhu *et al.*, 2007; Driever *et al.* 2017) and thus a bioengineering approach is required, with proof of concept emerging (Lopez-Calcagno *et al*., 2020).

The promise of simultaneously enhancing RuBP regeneration and electron transport capacity was demonstrated recently by the introduction of a cyanobacterial bifunctional enzyme fructose-1,6-bisphosphatase/SBPase or the overexpression of the plant enzyme SBPase together with the expression of the red algal protein cytochrome *c*_6_ in tobacco (Lopez-Calcagno *et al*., 2020). The engineered plants had enhanced photosynthesis and water use efficiency, and produced more biomass.

In C_3_ plants such as wheat, Rubisco catalyses approximately two oxygenations for every five carboxylations at contemporary levels of atmospheric CO_2_ and temperature (Walker *et al.*, 2016). Considering the CO_2_ and NH_4_^+^ losses and energy expense during the photorespiratory cycle, Walker *et al.* (2016) estimated that photorespiration decreases wheat yields in the USA by 20% and showed that decreasing photorespiration relative to photosynthesis would lead to significant economic gains. This could be achieved through large increases in the concentration of CO_2_ (relative to O_2_) in the vicinity of Rubisco via introduction of a carbon-concentrating mechanism such as those present in cyanobacteria, green algae, and plant species with C_4_ or C_2_ photosynthesis (Lundgren, 2020). Alternative photorespiratory pathways have also shown promise in lowering the cost of this process in model species (South *et al.*, 2018).

### Photoinhibition and photoprotection (Link 7)

Excessive light energy is relatively common and can inactivate photosystem reaction centres and induce the formation of reactive oxygen. These are well regulated by the plant but the former (sometimes termed photoinhibition) can reduce photosynthesis in low light, sometimes to an extent that causes loss of RUE and productivity (Burgess *et al*., 2015; Hubbart *et al.*, 2018). Photoprotection refers to a suite of processes that help to prevent or reduce these effects, and one of the most common (NPQ) is so prevalent that it can reduce quantum yield in low light too, a common occurrence. Both photoprotection and photoinhibition have long been predicted to be limiting to biomass and yield, since they determine leaf-level quantum yield (most leaves in a canopy will be light limited and light saturated in turn), but empirical data were lacking. Recent work in tobacco and soybean showed that by accelerating the recovery from photoprotection using specific and known genes, such as those encoding PsbS and zeaxanthin epoxidase, it was possible to limit this loss and enhance biomass production (Kromdjik *et al*., 2016; De Souza *et al.*, 2022). Enhancement of photoprotection alone by increasing capacity for PsbS resulted in greater biomass and yield in rice (Hubbart *et al.*, 2018).

Natural genetic variation for NPQ induction and relaxation can be found in wheat genotypes and wheat wild relatives, suggesting that a breeding approach may be possible for improvement (McAusland *et al.*, 2020), and in rice (Cowling *et al.*, 2022). In a similar way to Rubisco capacity and activation state, a canopy-dependent strategy may be necessary for further optimization since the capacity for protective NPQ seems to be greater in the lower, shaded, regions of the canopy where it is required for enhancement of photoprotection, as shown for rice (Foo *et al.*, 2020).

### Leaf structure and capacity, and CO_2_ diffusion (Link 8)

Leaf capacity for photosynthesis can refer to the concentration of photosynthetic components per unit leaf area within an optimized specific leaf weight (leaf thickness). As such it is highly correlated with N per unit leaf area. However, the internal structure of the leaf has key 3D properties and biophysical characteristics that influence photosynthesis efficiency, namely the exposed mesophyll cell surface area, cell density, and gas space volume for efficient gas transfer. One of the key features and measurements is the mesophyll conductance or chloroplast conductance value which is determined by the efficiency of gas transfer from the internal gas spaces to the sites of carboxylation. This is correlated with photosynthesis in wheat, and genetic variation exists for these conductances (Jahan *et al.*, 2014; Lundgren *et al.*, 2019). Cell density and airspace patterning have been considered to be important in the improvement of intra leaf conductance (Lehmeier *et al.*, 2017), but progress remains to be made in completely understanding the genetic regulation of mesophyll tissue development in leaves (Terashima *et al.*, 2011; Tholen *et al.*, 2012; Lundgren *et al.*, 2019). It is also worth pointing out that the structure of non-foliar organs with respect to photosynthetic capacity, regulation, and constraints to gas diffusion (along with the source of CO_2_) seems to remain poorly understood despite its importance (Simkin *et al.*, 2020).

### Stomata properties (Link 9)

Stomata are one of the most important organs in the plant, gating the exchange of CO_2_ and water between the internal leaf and the external environment. Key to water use efficiency trade-offs, they limit the availability of CO_2_. There are two important properties: their physical determination of gas flux rates and the speed with which they respond to changes in the environment. Research across species including wheat has shown that stomatal density can be reduced with no effect on photosynthesis but an improvement in water use efficiency (Lawson and Blatt, 2014; Hughes *et al.*, 2017). Stomata with faster opening and closing should improve both dynamic photosynthesis and water use efficiency, with a metabolic cost. When water is not limiting, stomatal characteristics also have a major impact on plant operating temperature by regulating the evapotranspiration rate (Amani *et al.*, 1996).

In wheat, stomata respond quickly to an increase in light and continue to open after near maximum CO_2_ assimilation is reached (McAusland *et al.*, 2016). This overshooting of stomatal conductance decreases water use efficiency, and is predicted to be important especially in the vegetative stage; saving water at this stage by making stomata more efficient could save water to support grain filling later on. A comparison of eight European wheat cultivars grown under controlled conditions showed variation for the speed of stomatal opening across cultivars and with leaf age, and a good correlation to photosynthesis, with genes such as Blue Light Signalling 1 (TaBLUS1) controlling stomatal aperture in response to light (Faralli *et al.*, 2019a, b).

### Respiratory metabolism (Link 10)

Dark mitochondrial respiration is a major primary process, responsible for processing a very large proportion of photosynthesis-derived carbohydrate to generate ATP, reducing power, and metabolic precursors. In doing so, it drives growth of all plants, and therefore variation in efficiency of respiration can determine plant-level energy use efficiency and therefore yield in an analogous way to the arguments made for photosynthesis above (Posch *et al.*, 2019). Genetic variation for dark respiration in wheat has been shown (Scafaro *et al.*, 2017; Coast *et al.*, 2019). Methods for accurately measuring dark respiration are problematic since they require excision of all types of tissue including roots. Nonetheless evidence has been presented for enhanced photosynthesis and productivity in plants with reduced respiration rates (e.g. Nunes-Nesi *et al.*, 2005), and the genetic basis in cereals is being elucidated (e.g. Qu *et al.*, 2020). It has been proposed that enhanced respiration, especially at night, may deplete carbohydrate reserves and prevent their contribution to yield (Xu *et al.*, 2021), but this is not always the case (Peraudaeu *et al*., 2015).

Respiration is highly sensitive to various environmental components especially temperature, and is metabolically linked with photosynthesis. High temperatures initially induce higher rates of cellular respiration, commonly followed by thermal acclimation whereby the tissue achieves homeostasis according to energy supply and demand for growth and maintenance (Yamori *et al.*, 2014), but it is unclear how this affects wheat source productivity or yield (Posch *et al.*, 2019). Recent work with rice indicated that increased nocturnal respiration was associated with depletion of non-structural carbohydrates (Xu *et al.*, 2021). High-throughput screening will prove valuable for understanding the genetic basis of respiratory responses. A high-throughput remote-sensing method that models hyperspectral data has been shown to be associated with dark respiration and provides evidence for genetic variation in this process (Coast *et al.*, 2019).

## Root capacity and function (Link 11)

Roots are obviously an essential component of plant form and function, and they provide means to capture soil water and essential mineral elements needed to generate a canopy to provide photosynthate. They also form intricate growth-promoting interactions with microorganisms in the soil and are the means by which many endophytes enter plants to colonize plant tissues (de Vries *et al.*, 2020). Root properties are rarely measured in experiments involving yield components, and their role in generating RUE, whilst self-evident, is quantitively unclear since it is aboveground dry matter that is most commonly measured. Therefore, variation in root growth may represent a source of genetic improvement of RUE, but it is not clear how this will interact with soil resource acquisition in different environments (Murchie and Reynolds, 2012). Soils are complex: root system properties such as architecture (depth, root front velocity, and root angle) could be improved in suboptimal conditions to enhance capture, especially under conditions where water, essential microbes, or nutrients are limiting or partially limiting (Manschadi *et al.*, 2006; Ober *et al.*, 2021). In yield potential conditions, it is conceivable that the same properties may be of benefit and may influence post-anthesis events such as stay green and N remobilization (Foulkes *et al.*, 2016; Nehe *et al.*, 2018). These include seminal root number, root hairs, and total root length for which QTLs have been discovered (Horn *et al.*, 2016; Xie *et al.*, 2017; Soriano and Alvaro, 2019). The penetration and vascular capacity of the root system can also have a large impact on the operating temperature of transpiring tissue aboveground (i.e. the canopy temperature, which is typically several degrees below ambient under well-watered conditions) (Lopes and Reynolds, 2010).

## Interactions: non-grain sink organs and processes common to source and grain sinks (Link 12)

As mentioned at the start of this review, plant growth rate is by definition tuned to the activities of both the source and sink. In general, the two should be in ‘balance’ such that an enhancement of one can induce an enhancement of the other, within developmental limitations. Therefore, an understanding of the coordination of source and sink interactions and signalling during conditions that can affect the strength of either is important. Experiments that have manipulated source or sink have clearly shown control acting in both directions. For example, partial defoliation results in enhancement of photosynthetic activity in the remaining leaves, demonstrating that a high sink to source ratio can lead to up-regulation of the source, but this can depend on growth stage (G.X. Zhu *et al.*, 2004; White *et al.*, 2016; Rivera-Amado *et al.*, 2020). Indeed, the introgression of *Rht* genes during the green revolution increased the post-anthesis sink to source strength ratio, and increased RUE clearly during post-anthesis but not in pre-anthesis (Miralles and Slafer, 1997). Genetic (7Ag.7DL translocation) as well as light treatments during grain set, both of which increased sink strength compared with checks, boosted the flag leaf light-saturated photosynthetic rate by ~10% when measured during grain filling (see table 3 in Reynolds *et al.*, 2009). Sink reduction can also lower leaf photosynthetic activity in wheat (Rivera-Amado *et al.*, 2020). Enhancing the source capacity with elevated CO_2_ has been used to show that a high sink strength (in roots, leaves, or shoots) helps to prevent the down-regulation of photosynthesis (Ruiz-Vera *et al*., 2017, 2021; Torralbo *et al.*, 2019). Overall, enhanced photosynthesis seems capable of driving yields higher where there is sufficient sink capacity, but the increased yields are still less than expected from photosynthesis alone (Ainsworth and Long, 2021). This would seem to indicate a need to improve both source and sink and their interactions in order to maximize yield improvement (Reynolds *et al.*, 2022).

The internal factors that regulate the feedforward and feedback processes are reasonably well understood, with some of the molecular players known (Lawlor and Paul, 2014; Paul *et al.*, 2020). Metabolic control of source activity begins within the leaf whereby the accumulation of hexose sugars represses the export from the chloroplast and the expression of photosynthesis (Paul and Foyer, 2001; Smith and Stitt, 2007). It has been proposed that the glucose sensor hexokinase, the TOR protein kinase signalling pathway, the protein kinase SnRK1, and the regulatory metabolite trehalose-6-phosphate (T6P) all act to regulate source–sink activity and thereby influence plant growth (Smeekens *et al.*, 2010; Lastdrager *et al.*, 2014; Meitzel *et al.*, 2021). T6P is thought to be essential for carbohydrate signalling and regulation, and acts as an inhibitor of the ‘feast or famine’ protein kinase SnRK1. Increased levels of sucrose (mainly) in the plant stimulate T6P synthesis, de-repressing the activity of pathways involved in growth and development via gene expression (Nunes *et al.*, 2013). The activity of the T6P pathway according to sucrose level depends on tissue and developmental stage (Martinez-Barajas *et al*., 2011). This provides a means of understanding at a molecular level how source–sink signalling might occur, and has been studied in several species including wheat (Paul *et al.*, 2020). Wheat grains show differences in T6P content during development, with evidence that high levels may be associated with increased grain size and sink strength (Griffiths *et al.*, 2016; Paul *et al.*, 2017). T6P is also involved in the responses to environmental stress in wheat, such as enhancement of growth following the recovery after drought (Griffiths *et al.*, 2016). Other approaches include understanding further the role of α-expansins that appear to limit the size of expanding grain (Lizana *et al.*, 2010). Overexpressing α-expansin using a wheat transgenic approach was shown recently to influence grain size and yield without the usual trade-off in grain number (Calderini *et al.*, 2021). This approach improved yield by >10% through increasing grain size with little impact on grain number.

In wheat, the stem tissue plays an important role in regulating whole-plant source–sink interactions by providing a temporary but substantial sink for carbohydrate and nitrogen. Substantial amounts of carbohydrate (in the form of WSCs, predominantly fructans and minor components of sucrose, fructose, and glucose) are stored within the stems and remobilized post-anthesis to provide fixed carbon for grain filling (Wardlaw and Willenbrink, 1994; Xue *et al.*, 2008). There is some evidence that stem upper internodes tend to accumulate WSCs more rapidly once the demands for spike growth are fulfilled (Bonnett and Incoll, 1992; Gebbing, 2003), suggesting that spikes may be the priority sink for assimilate accumulation in the rapid spike growth phase during stem elongation. The underpinning biochemical mechanisms governing source–sink regulation including sensing of carbohydrates and subsequent allocation to stems and grains are still largely undetermined but nevertheless crucial for deposition of carbohydrate for grain yield (grain number and size; Paul *et al.*, 2020). In addition, interactions of phytohormones with factors such as those involved in sugar signalling and N status play an important role in regulating source and sink communication (Paul and Foyer, 2001; Thomas and Ougham, 2014).

The proportion of final grain carbohydrate that is made up by temporary stem reserves is genotype and environment dependent. For example, heat stress and drought during the grain-filling phase reduce current photosynthesis and increase reliance of yield on stored carbohydrate (Blum *et al.*, 1994; Wang *et al.*, 2012). It is also the case that these reserves act as a sink and probably reduce sink limitation of photosynthesis during this phase. There is well known genetic variation in the capacity of the stem to store carbohydrates (Ruuska *et al.*, 2006; Snape *et al.*, 2007; Saint Pierre *et al.*, 2010; McIntyre *et al.*, 2012). Although a mechanistic relationship still lacks direct evidence, the capacity of the stem to accumulate WSCs has been correlated with yield in wheat, and QTLs have been identified (Snape *et al.*, 2007; Zhang *et al.*, 2008). Variation in WSC content has been discovered to be mainly due to fructan (Ruuska *et al.*, 2006). Many recent studies conclude that the genetic basis for WSC capacity is still unclear (Li *et al.*, 2020) although recent genome-wide association studies have provided genetic markers (Fu *et al.*, 2020). Interestingly, breeding for elevated WSC concentration resulted in fewer tillers and less grain per m^2^ but higher harvest index (Rebetzke *et al.*, 2008). The interplay with nitrogen supply in this process also needs further attention (Zahedi *et al.*, 2004).

Increasing ambient temperatures, frequencies of heatwaves, and reduced water availability during end of season grain filling pose significant threats to grain yield (size and grain number). Recently, Barrero *et al.* (2020) demonstrated that variability exists in the capacity of wheat genotypes to be resilient to a heat event during grain filling, with grain size not impacted. During this phase, it has been shown that deposition of carbohydrate within the grain is impaired at elevated temperature (Jenner, 1991); however, increasing the duration of flag leaf photosynthesis seems to have no impact on allocation of carbohydrate to grains during filling (Borrill *et al.*, 2015).

Any comprehensive strategy to improve wheat yield potential must include lodging resistance. For example, tiller production will affect the lodging risk, with higher tiller number per plant leading to decreased stem strength and root anchorage of individual tillers, which increases risk of stem and root lodging, respectively. The risk of stem and root lodging will also be related to stem–internode and root anchorage traits affecting stem lodging and root lodging, respectively (Pinera-Chavez *et al*., 2016).

## Summary and conclusion

We have here provided a summary and rationale for source activity components that exist in wheat and we have placed them in a context of developmental phases and the formation of sink tissue (Slafer *et al.*, 2023). The evidence assembled provides support for the WDs that emphasize the links between processes and activities and agricultural yields. The review emphasizes the increasing need to recognize that the photosynthesis improvements, whilst needed to provide the extra biomass to raise yield, need to be considered within the context of (i) the complexities of canopies, vertical variation in light captures, and the multiple photosynthetic sources including spikes; and also (ii) the plant requirements which in wheat are the optimal formation and filling of the major sinks: the grain and the stem pre-heading storage cells. Such considerations are essential if we are to place source improvements into the correct context and provide accurate paramaterizations for prediction of their role in crop yield formation, together with the relevant genes, such as the recent examples demonstrate.
